# BCAA supplementation enhances milk fat synthesis in Yili mares and promotes foal growth through remodeling of intestinal amino acid metabolism

**DOI:** 10.3389/fmicb.2025.1699614

**Published:** 2025-11-06

**Authors:** Chen Meng, Yaqi Zeng, Jianwen Wang, Xinkui Yao, Jun Meng

**Affiliations:** 1 College of Animal Science, Xinjiang Agricultural University/Equine Industry Research Institute, Xinjiang Agricultural University, Urumqi, Xinjiang, China; 2 Xinjiang Key Laboratory of Equine Breeding and Exercise Physiology, Urumqi, Xinjiang, China

**Keywords:** mares, mare milk, branched-chain amino acids, foals, fecal microbiota

## Abstract

**Introduction:**

This study evaluated the effects of graded supplementation of branched-chain amino acids (BCAAs) in lactating mares on lactation performance, foal growth, and metabolic responses.

**Methods:**

Twenty mare-foal pairs were assigned to control, low- (38 g/d), medium- (76 g/d), or high-dose (114 g/d) groups. Milk and blood samples were collected over 60 days for composition, hormone, and metabolomic analyses. Fecal microbiota from the foals was also examined.

**Results:**

BCAAs supplementation interacted with lactation stage, enhancing milk fat yield and increasing milk growth hormone and progesterone. The medium dose (76 g/d) was effective, while 114 g/d showed the strongest effects. High-dose BCAAs altered organic acid abundance, influencing lipid, energy, and BCAA metabolism, correlating with milk composition changes. In foals, altered milk reduced serum BCAAs and other amino acids but elevated growth hormones (GH, INS, IGF-1) dose-dependently. Antioxidant and immune parameters were unaffected. The high dose increased blood urea nitrogen, indicating higher nitrogen load, whereas the medium dose supported growth without metabolic stress. Fecal microbiota analysis revealed enriched amino acid degradation pathways, especially for BCAAs.

**Discussion:**

We conclude that BCAAs supplementation regulates milk fat synthesis and promotes foal growth via a milk–microbiota–metabolism axis, providing a basis for improving milk quality and offspring development through maternal nutrition.

## 1 Introduction

Branched-chain amino acids (BCAAs), including leucine (Leu), isoleucine (Ile), and valine (Val), are essential hydrophobic amino acids characterized by their branched aliphatic side chains (Bo and Fujii, 2024). Since mammals cannot synthesize BCAAs de novo, they must be obtained through dietary intake (Green et al., 2024), whereas most bacteria, fungi, and plants are capable of endogenous synthesis (Hai et al., 2024).

In addition to serving as precursors for nitrogen-containing compounds, BCAAs participate in both ketogenic and glucogenic pathways and thus play regulatory roles in carbohydrate, lipid, and protein metabolism (Mansoori et al., 2025). They also contribute to maintaining the balance between pro- and anti-inflammatory cytokines, and exert immunomodulatory, antioxidant, and protective effects (Gallagher et al., 2024; Choi et al., 2024). Previous studies have shown that exogenous BCAAs supplementation can enhance lactational performance by increasing milk yield as well as milk protein and fat contents (Ren et al., 2025; Wang X. et al., 2025; Huang et al., 2024; Lu et al., 2024). In terms of immunity, BCAAs strengthen host defenses by regulating cellular, humoral, and mucosal immune responses (Qin et al., 2025; Xiong et al., 2025; Ahmad et al., 2024; Zheng et al., 2025), while dietary deficiency reduces lymphocyte and leukocyte numbers, thereby impairing innate immune function (Lu et al., 2024). Moreover, BCAAs exhibit strong antioxidant activity, both by scavenging free radicals and by enhancing antioxidant enzyme activity (Zheng et al., 2025; Bifari et al., 2017). They also provide an important energy source for intestinal nutrient transport and intracellular protein turnover (Bo and Fujii, 2024), while modulating the expression of nutrient transporters and supporting gut barrier integrity (Gojda and Cahova, 2021; Wang H. et al., 2025; Yan et al., 2022; Zhang et al., 2017). Increasing evidence has further highlighted a bidirectional interaction between BCAAs and the gut microbiota, offering new perspectives on their multifaceted roles.

Most studies on BCAAs and lactation have focused on dairy cows and sows, with limited evidence available in equine species. In mares, maternal nutritional status directly affects milk production and composition, which in turn influences the growth and health of suckling foals. Optimizing BCAAs intake in lactating mares through dietary strategies therefore represents a safe and effective approach to improving milk yield and supporting foal development. Based on this rationale, the present study investigated the effects of three levels of BCAAs supplementation in Yili mares on milk yield and composition, milk hormone profiles, foal serum hormones, antioxidant and immune function, amino acid metabolism, and fecal microbiota diversity. The findings aim to provide a theoretical basis for advancing the application of BCAAs in equine nutrition.

## 2 Materials and methods

### 2.1 Experimental design and sampling

**Figure 1:**
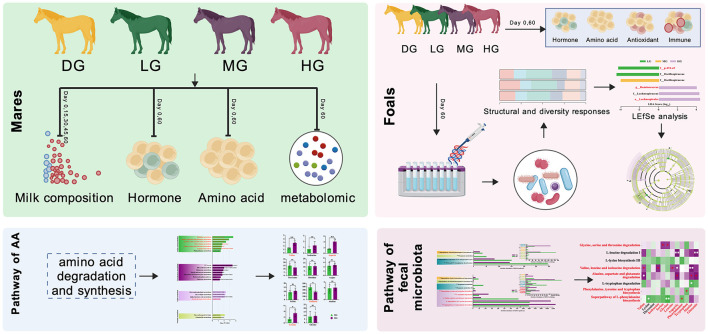
Experimental design.

The experiment was carried out at Zhaosu Horse Farm, Yili Kazakh Autonomous Prefecture, Xinjiang, China. Twenty lactating Yili mares and their suckling foals (2 ~ 3 months of age) were enrolled. The initial body weights were 97.60 ± 13.24 kg for foals and 392.90 ± 12.18 kg for mares. Foals were randomly assigned to four groups (*n* = 5 per group): control (DG), low-dose (LG), medium-dose (MG), and high-dose (HG). Mares were grouped together with their respective foals (Figure 1).

All groups were managed under identical conditions for 67 days, including a 7-day adaptation period followed by a 60-day feeding trial. During the trial, each mare was provided with 2 kg of concentrate feed daily (ingredient composition shown in Table 1). In addition, mares in the treatment groups received a BCAA mixture (Ile:Leu:Val = 1:2:1.2; Xinjiang Lianying Biotechnology Co., Ltd.) at the following doses: DG, 0 g/d; LG, 38 g/d; MG, 76 g/d; and HG, 114 g/d.

**Table 1:** Composition and nutrient level of concentrate supplement (DM basis).

**Ingredients**	**Content (%)**	**Nutrient components^b^**	**Content (%)**
Corn	46	DM	90.76
Barley	8	CP	14.44
Wheal bran	8	NDF	61.68
Raoeseed meal	28	ADF	9.95
Premix^a^	10	Ca	0.63
Total	100	P	0.35

^a^Premix provided per kilogram of concentrate: Vitamin A 120,000 IU; Vitamin D3 30,000 IU; Vitamin E 2,500 IU; Niacin 300 mg; Copper 250 mg; Iron 1,200 mg; Zinc 1,200 mg; Manganese 1,100 mg; Iodine 8 mg; Selenium 6 mg; Cobalt 4 mg.

^b^Nutrient contents are measured values.

Routine management practices were maintained throughout the study. Each morning at 10:00, mares and foals were brought from pasture to the milking area. After separation, mares were moved to individual stalls and fed their assigned BCAAs dose, mixed with a small volume of water and blended into the 2 kg concentrate ration. Milking was performed four times daily at 1.5-h intervals (11:00, 12:30, 14:00, and 15:30). During milking, foals remained separated with free access to water and ample forage. After the final milking session, mares and foals were reunited and returned to pasture for grazing.

### 2.2 Mare milk sample collection

Milk samples were collected on days 0, 15, 30, 45, and 60 of the trial. Milk yield for each mare was recorded using a spring scale. From each of the four daily milking sessions, 25 mL was collected, pooled, and transferred into a 100 mL sample bottle, then immediately stored at −20°C for milk composition analysis. On day 60, an additional 5 mL of the pooled sample was transferred into a cryovial, flash-frozen in liquid nitrogen for 15 min, and stored at −80°C for targeted amino acid quantification and untargeted metabolomic analysis.

### 2.3 Foal blood sample collection

On days 0 and 60, foals were separated from their dams and fasted for 2 h before 10 mL of blood was collected from the jugular vein into vacuum serum tubes. Samples were allowed to clot at room temperature for 1 h and then centrifuged at 3,500 rpm for 15 min. The supernatant was aliquoted into 2 mL tubes, labeled, and stored at −20°C for analysis of hormones, immune and antioxidant parameters, and targeted amino acids.

### 2.4 Foal fecal sample collection

On day 60, rectal fecal samples (~ 5 g) were collected from all 20 foals, placed in 5 mL cryovials, immediately flash-frozen in liquid nitrogen, and then transferred to −80°C for 16S rRNA sequencing.

### 2.5 Milk composition analysis

Frozen milk samples were thawed in a 40°C water bath and analyzed with a MilkoScan^TM^ FT3 analyzer (Foss Electric, Hillerød, Denmark). After 30 min of preheating and calibration, milk fat, lactose, protein, casein, glucose, and other components were measured.

### 2.6 Milk hormone and free amino acid assays

Commercial ELISA kits were used to measure prolactin (PRL), growth hormone (GH), insulin (INS), progesterone (PROG), and estradiol (E_2_), following the manufacturers' protocols. The ELISA kits were purchased from Wuhan Huamei Bioengineering Co., Ltd. (Shenzhen, China), with the following catalog numbers: PRL (CSB-EL018724HO), GH (CSB-E11242Eq), INS (CSB-EL011742HO), PROG (CSB-E13183Hs), and E2 (CSB-EQ027953HO). According to the manufacturer's datasheets, the detection ranges were as follows: PRL (2.5 – 1000 ng/mL), GH (25 – 400 ng/mL), INS (1.56 – 100 nIU/mL), PROG (0.25 – 100 ng/mL), and E2 (50 – 1200 pg/mL). The intra-assay and inter-assay coefficients of variation were both < 15%. Targeted amino acid profiling including alanine (Ala), arginine (Arg), asparagine (Asn), aspartic acid (Asp), creatinine (Cr), glutamine (Gln), glutamic acid (Glu), glycine (Gly), histidine (His), isoleucine (Ile), leucine (Leu), lysine (Lys), methionine (Met), ornithine (Orn), phenylalanine (Phe), proline (Pro), serine (Ser), threonine (Thr), tryptophan (Trp), tyrosine (Tyr), and valine (Val) was conducted by Novogene Co., Ltd. (Tianjin, China) using LC-MS/MS.

### 2.7 Untargeted milk metabolomics analysis

Metabolites were separated using a Vanquish UHPLC system (Thermo Fisher Scientific) equipped with a Hypersil GOLD C18 column at 40°C and 0.2 mL/min. For positive ion mode, mobile phase A was 0.1% formic acid in water and phase B was methanol; for negative ion mode, mobile phase A was 5 mmol/L ammonium acetate in water and phase B was methanol. To enable comprehensive interpretation of the identified metabolites, functional annotation was performed using multiple public databases, including the Kyoto Encyclopedia of Genes and Genomes (KEGG; https://www.genome.jp/kegg/pathway.html), the Human Metabolome Database (HMDB; https://hmdb.ca/metabolites), and LIPID Maps (http://www.lipidmaps.org/).

Metabolomics data were preprocessed using the metaX tool, followed by multivariate statistical analysis. For unsupervised analysis, PCA was applied to assess the natural clustering tendencies among sample comparisons. For supervised analysis, orthogonal partial least squares discriminant analysis (OPLS-DA) was employed to calculate Variable Importance in Projection (VIP) scores, and metabolites with VIP > 1 were retained for further analysis. Univariate analysis was performed using Student's *t*-test (*P* < 0.05) to identify statistically significant differences between comparisons. Metabolites meeting both criteria (VIP > 1 and *P* < 0.05) were considered significantly differential and subjected to KEGG pathway enrichment analysis using MetaboAnalyst (https://www.metaboanalyst.ca/, accessed November 24, 2024).

### 2.8 Foal serum parameter assays

Antioxidant indices, including hydroxyl radical (-OH), superoxide dismutase (SOD), total antioxidant capacity (T-AOC), malondialdehyde (MDA), glutathione peroxidase (GSH-PX), and catalase (CAT), as well as hormone indicators including GH, somatostatin (SS), INS, and IGF-1, were measured using a microplate reader (DR-200DS, HuavideLang) and corresponding commercial kits. Immune indices, including immunoglobulin A (IgA), immunoglobulin G (IgG), total protein (TP), albumin (ALB), globulin (GLB), and blood urea nitrogen (BUN), were measured using an automated biochemical analyzer (BA200). Targeted amino acid analysis was performed by Novogene Co., Ltd. (Tianjin, China) using LC-MS/MS.

### 2.9 Foal fecal microbiota 16S rRNA sequencing and bioinformatics

Genomic DNA was extracted from fecal samples and amplified using primers targeting the V3 ~ V4 region of the 16S rRNA gene (341F: CCTAYGGGRBGCASCAG; 806R: GGACTACHVGGGTWTCTAAT). PCR products were verified on 2.0% agarose gels, purified, quantified, and pooled in equimolar ratios for library construction. Libraries were sequenced on a NovaSeq 6000 platform (PE250). Raw reads were processed to generate high-quality clean tags through demultiplexing, merging, quality filtering, and chimera removal. ASVs were generated in QIIME2 and assigned taxonomies against the Silva database. Alpha diversity (Chao1, Shannon) and beta diversity (PCoA) were assessed. Differential taxa were identified with LEfSe. Functional predictions were made using PICRUSt2 against KEGG. Significantly enriched pathways were defined as |log_2_ fold change| ≥ 1 and *P* < 0.05.

### 2.10 Statistical analysis

Longitudinal data (milk yield, hormone, antioxidant, and immune parameters) were analyzed using linear mixed-effects models (LMM) in R (v4.4.3, lme4 package). Fixed effects included BCAA treatment, time, and their interaction; mare ID was included as a random effect. *P*-values were obtained using lmerTest. Estimated marginal means were calculated using emmeans, with Tukey's HSD and multcomp for multiple comparison correction. Visualization was performed with ggplot2. Pearson correlations were computed in RStudio, with |*r*|>0.7 and *P* < 0.05 considered strong. Heatmaps were generated with ggplot2. Between-group differences in amino acids, hormones, antioxidant/immune markers, metabolites, and microbial functions were tested using Student's *t*-test or one-way ANOVA in GraphPad Prism (v9.4.1). Significance was set at ^ns^*P* > 0.05; ^*^P < 0.05; ^**^P < 0.01.

## 3 Results

### 3.1 Dose-dependent responses of milk fat yield and milk hormones to BCAAs supplementation

**Figure 2:**
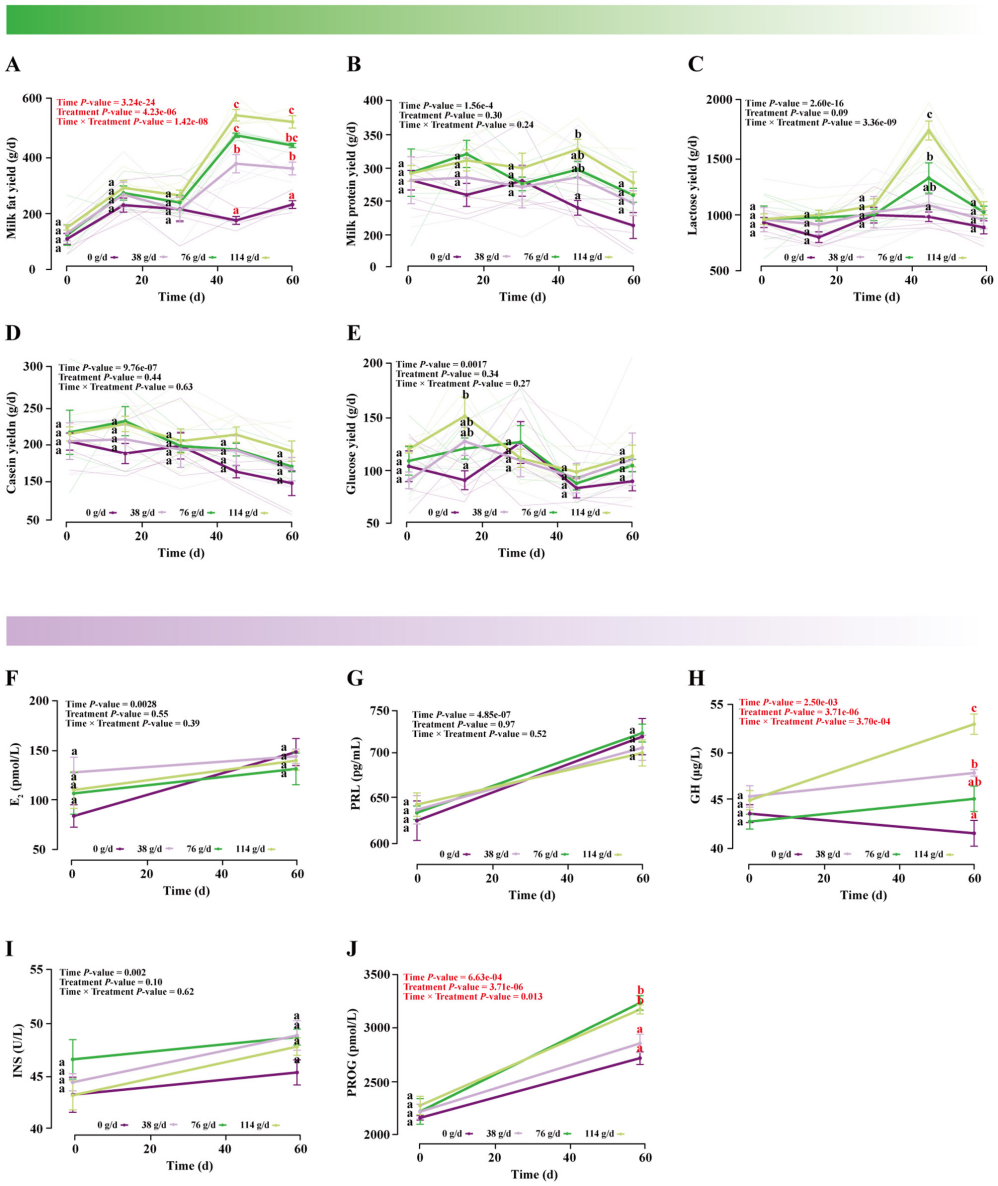
Effects of dietary supplementation with different doses of BCAAs on lactation performance indicators. A linear mixed model was used to analyze the interaction effect between BCAA treatment (Treatment) and time (Time). **(A)** Milk fat yield, **(B)** milk protein yield, **(C)** lactose yield, **(D)** casein yield, **(E)** glucose yield, **(F)** E_2_ concentration in mare milk, **(G)** PRL concentration in mare milk, **(H)** GH concentration in mare milk, **(I)** INS concentration in mare milk, **(J)** PROG concentration in mare milk. Data were compared using Tukey's HSD test. Values marked with the same letter are not significantly different (*P* > 0.05). Different lowercase letters indicate a significant difference (*P* < 0.05), and different uppercase letters indicate a highly significant difference (*P* < 0.01).

LMM analysis revealed that the interaction between BCAAs dose and lactation time had no significant effects on milk protein, lactose, casein, or glucose yields, nor on E_2_, PRL, or INS levels (*P* > 0.05; (Figures 2B–G, I). In contrast, significant effects were observed for milk fat yield (Treatment × Time: *F*(12.00, 63.99) = 7.67, *P* = 1.42e-08), GH (Treatment × Time: *F*(3.00, 31.99) = 8.10, *P* = 3.70e-04), and PROG (Treatment × Time: *F*(3.00, 31.99) = 4.16, *P* = 0.01) (Supplementary Table S1).

Post-hoc Tukey's HSD tests further indicated that medium- and high-dose BCAAs groups exhibited a marked increase in milk fat secretion starting from day 30, reaching a peak at day 45. Although a slight decline was observed thereafter, fat yields in these groups remained significantly higher than in low-dose and control groups at day 60, indicating sustained enhancement of milk fat metabolism (Figure 2A and Supplementary Table S2). A similar dose-dependent elevation was also observed for GH (Figure 2H and Supplementary Table S1) and PROG (Figure 2J and Supplementary Table S2). Integrated analysis suggested that 76 g/d was the threshold dose required to improve lactational performance, with the high dose showing the strongest effects.

### 3.2 Associations among milk hormones, amino acid profiles, and organic acid metabolites under BCAAs supplementation

**Figure 3:**
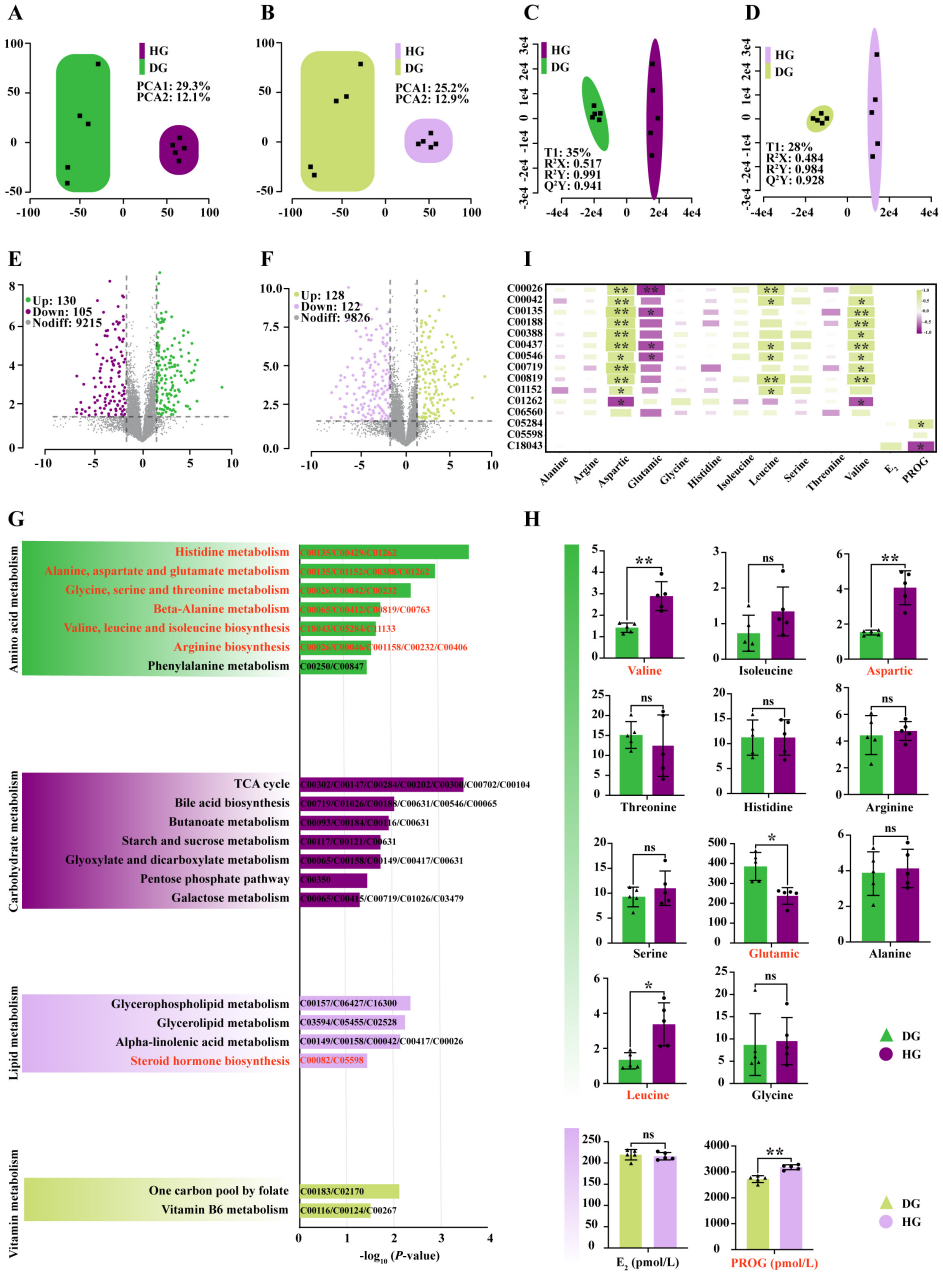
Comparative untargeted metabolomic analysis of mare milk between the high-dose BCAA group and the control group. **(A)** PCA score plot in positive ion mode. **(B)** PCA score plot in negative ion mode. **(C)** Permutation test results of the OPLS-DA model in positive ion mode. **(D)** Permutation test results of the OPLS-DA model in negative ion mode. **(E)** Volcano plot of DEMs in positive ion mode. **(F)** Volcano plot of DEMs in negative ion mode. **(G)** KEGG pathway enrichment analysis of DEMs. **(H)** Comparison of targeted amino acid content and hormone levels in mare milk between groups. Significance was determined by Student's t-test: ^ns^*P* > 0.05; *P < 0.05; **P < 0.01. **(I)** Pearson correlation heatmap between differentially abundant metabolites from key pathways and amino acids/hormones. ^ns^*P* > 0.05; *P < 0.05; **P < 0.01.

The PCA performed in both positive and negative ion modes showed clear separation between HG and DG groups, confirming adequate experimental grouping and supporting subsequent metabolomic analysis (Figures 3A, B). OPLS-DA permutation tests further confirmed high model fit and significance in both positive (R^2^Y = 0.99, *P* < 0.05) and negative (R^2^Y = 0.98, *P* < 0.05) ion modes (Figures 3C, D).

Using the criteria *P* < 0.05 and VIP > 1, 235 differentially expressed metabolites (DEMs) were identified in positive ion mode (130 upregulated, 105 downregulated; Figure 3E) and 250 DEMs in negative ion mode (128 upregulated, 122 downregulated; Figure 3F). Enrichment analysis indicated that BCAAs supplementation predominantly altered organic acids and derivatives (Figure 3G and Supplementary Table S3), thereby modulating amino acid, carbohydrate, lipid, and vitamin metabolism. Specifically, high-dose supplementation significantly affected three BCAAs (Val, Leu, Ile) and six free amino acids (His, Thr, Phe, Ala, Asp, Glu, Gly, Ser) in mare milk (Figure 3G). Among these, Leu (*P* < 0.05) and Val (*P* < 0.01) were significantly increased. For non-essential amino acids, Asp was elevated (*P* < 0.05), whereas Glu decreased (*P* < 0.05; Figure 3H). Altered metabolite abundance further impacted steroid hormone synthesis: PROG was significantly increased in the HG group (*P* < 0.01), whereas E_2_ showed no significant change (*P* > 0.05; Figure 3H).

Pearson correlation analysis (Figure 3I) revealed that Leu was positively correlated with α-ketoglutarate (*r* = 0.78, *P* = 0.0082), succinate (*r* = 0.73, *P* = 0.0156), N-α-acetyl-L-ornithine (*r* = 0.73, *P* = 0.0167), pyruvaldehyde (*r* = 0.72, *P* = 0.0293), D-glutamine (*r* = 0.82, *P* = 0.0041), and 3-methylhistidine (*r* = 0.72, *P* = 0.0280). Val was significantly correlated with succinate (*r* = 0.71, *P* = 0.023), L-histidine (*r* = 0.84, *P* = 0.002), DL-threonine (*r* = 0.79, *P* = 0.007), histamine (*r* = 0.75, *P* = 0.013), N-α-acetyl-L-ornithine (*r* = 0.78, *P* = 0.008), betaine (*r* = 0.70, *P* = 0.005), and D-glutamine (*r* = 0.81, *P* = 0.074). PROG was positively correlated with phenaceturic acid (*r* = 0.72, *P* = 0.033) and negatively correlated with cholesteryl sulfate (*r* = −0.75, *P* = 0.013).

### 3.3 Dose-dependent responses of suckling foal serum hormones, antioxidant, and immune indicators to maternal BCAAs supplementation

LMM analysis indicated that the interaction between maternal BCAAs dose and lactation time significantly affected foal serum GH (Treatment × Time: *F*(3.00, 16.00) = 4.56, *P* = 0.02), IGF-1 (Treatment × Time: *F*(3.00, 32.00) = 2.30, *P* = 0.04), INS (Treatment × Time: *F*(3.00, 32.00) = 14.80, *P* = 3.22e-06), and BUN (Treatment × Time: *F*(3.00, 16.00) = 2.61, *P* = 0.04), while other indicators remained unaffected (*P* > 0.05; Supplementary Table S4).

**Figure 4:**
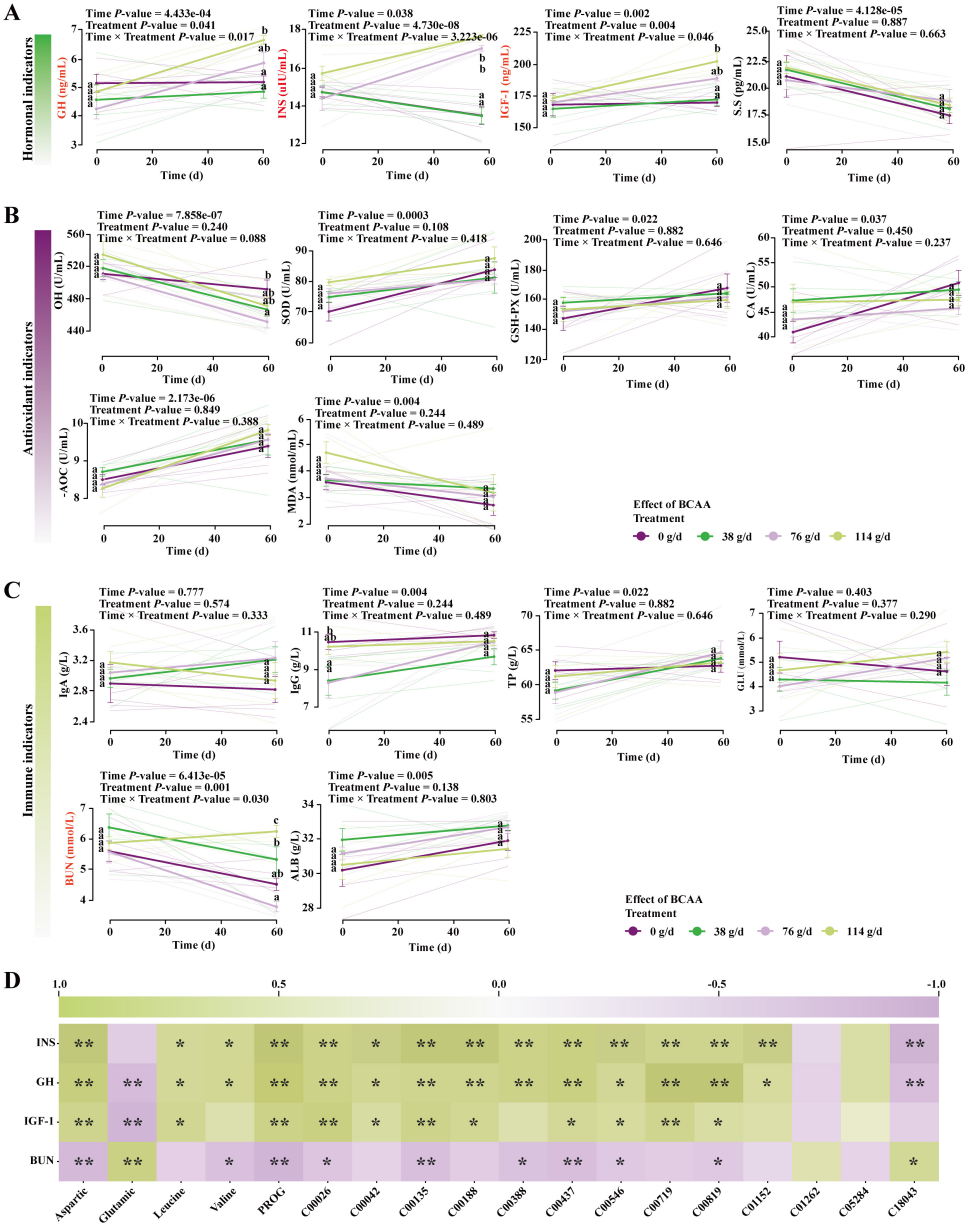
Effects of consuming milk with different doses of BCAAs on serum hormone levels, immune indices, and antioxidant parameters in foals. **(A)** Hormone levels, **(B)** antioxidant parameters, **(C)** immune indices. A linear mixed model was used to analyze the interaction effect between Treatment and Time. Group comparisons were performed using Tukey's HSD test. Values sharing the same superscript letter are not significantly different (*P* > 0.05). Different lowercase letters indicate a significant difference (*P* < 0.05), and different uppercase letters indicate a highly significant difference (*P* < 0.01). **(D)** Pearson correlation heatmap between significantly altered parameters in foals and the corresponding indicators in mare milk. ^ns^*P* > 0.05; *P < 0.05; **P < 0.01.

Tukey's HSD tests showed that foals consuming milk from medium- and high-dose mares exhibited significantly higher serum GH, IGF-1, and INS at day 60, with the strongest effects in the high-dose group (Figure 4A and Supplementary Table S5). BUN showed a non-linear dose response: although a cumulative effect was evident, foals in the HG group displayed significantly higher BUN, whereas those in the MG group had the lowest BUN (Figure 4C and Supplementary Table S5). Correlation analysis demonstrated significant associations between changes in foal serum indicators and milk-derived hormones, amino acids, and organic acids (Figure 4D).

### 3.4 Correlation between foal serum hormones, biochemical indicators, and serum amino acids

**Figure 5:**
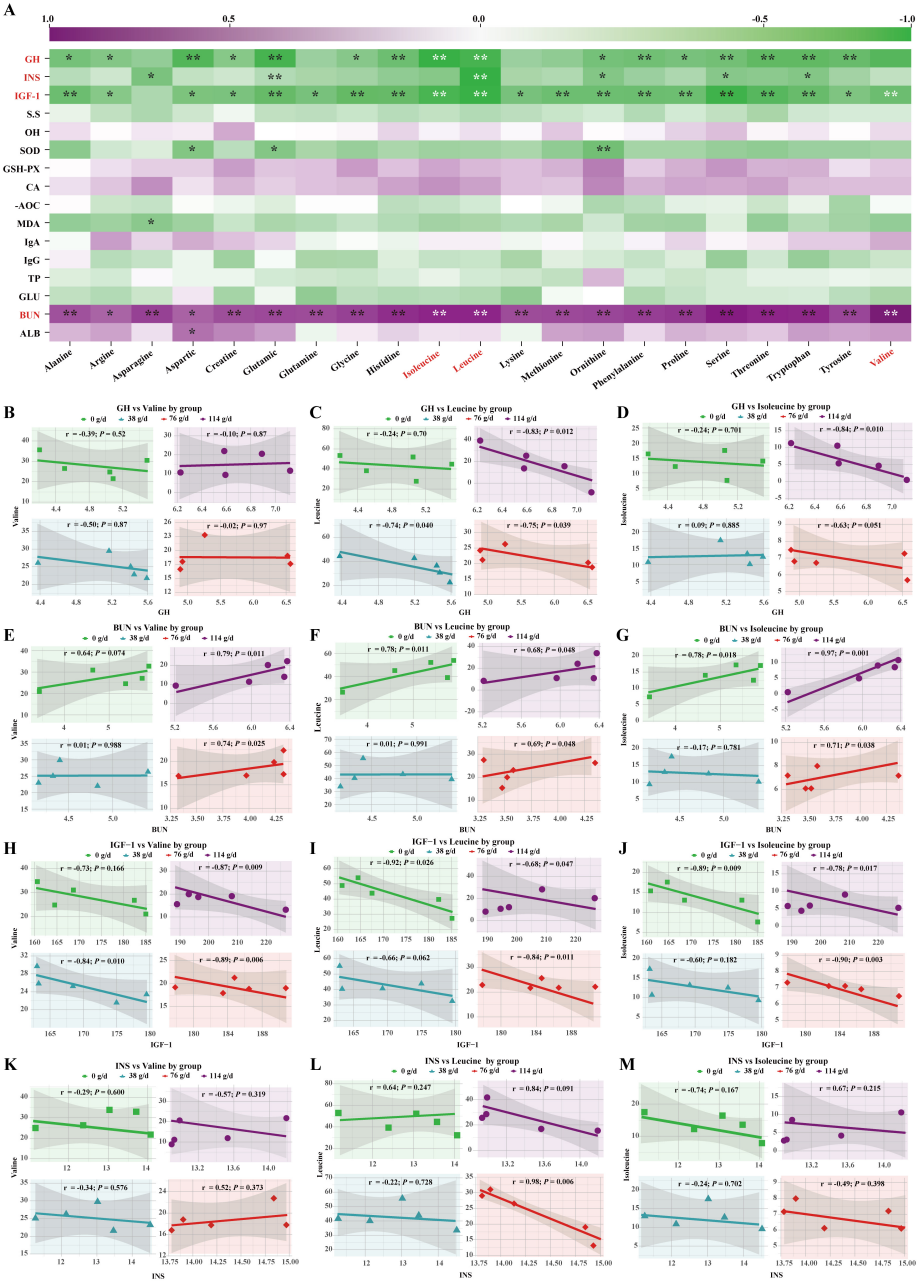
Correlation analysis between significantly altered parameters and serum AAs in foals. **(A)** Heatmap of Pearson correlation coefficients between all measured parameters and amino acids. **(B–D)** Scatter plots of the correlation between serum GH and BCAAs in each dose group. **(E–G)** Scatter plots of the correlation between blood BUN and BCAAs in each dose group. **(H–J)** Scatter plots of the correlation between IGF-1 and BCAAs in each dose group. **(K–M)** Scatter plots of the correlation between INS and BCAAs in each dose group.

Pearson correlation analysis revealed that foal serum GH was negatively correlated with amino acids, with the most pronounced negative correlations observed for the branched-chain amino acids (Ile and Leu) (Figure 5A). Among the groups, foals receiving high-dose BCAAs milk showed markedly stronger correlations compared with the other three groups (Figures 5C, D). Similarly, serum IGF-1 was negatively correlated with amino acids overall, with particularly significant negative correlations with branched-chain amino acids (Figure 5A). Although both the medium- and high-dose BCAAs milk groups displayed strong correlations, the medium-dose group exhibited a more pronounced effect (Figures 5H–J). In addition, serum INS was also negatively correlated with amino acids, showing a highly significant negative correlation with Leu (Figure 5A), and again the medium-dose group showed the strongest correlation (Figures 5K–M). In contrast, serum BUN was positively correlated with amino acids overall and showed highly significant positive correlations with branched-chain amino acids (Figure 5A). Notably, the correlation strength was significantly greater in the high-dose BCAAs milk group than in the other three groups (Figures 5E–G).

### 3.5 Structural and diversity responses of foal fecal microbiota to maternal BCAAs supplementation

**Figure 6:**
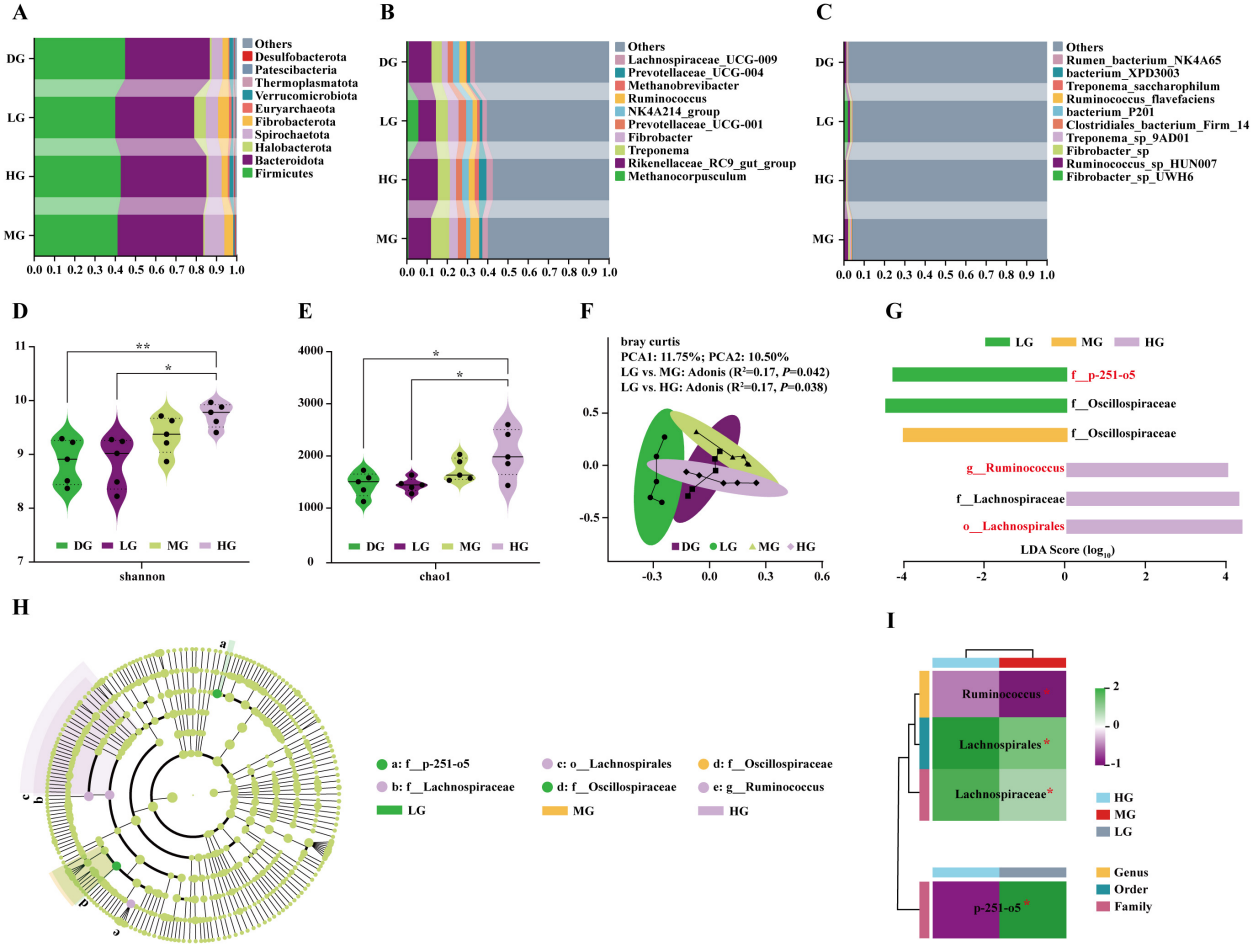
Effects of consuming milk with different doses of BCAAs on the structure and diversity of the foal fecal microbiota. **(A)** Microbial community composition at the phylum level. **(B)** Microbial community composition at the genus level. **(C)** Microbial community composition at the species level. **(D)** Comparison of alpha diversity (Shannon index) of the fecal microbiota among groups. **(E)** Comparison of alpha diversity (Chao1 index) of the fecal microbiota among groups. **(F)** PCoA based on Bray-Curtis distance. **(G)** LDA scores of key discriminative microbes identified by LEfSe analysis. **(H)** Cladogram showing the phylogenetic distribution of differentially abundant microbes. **(I)** Heatmap of the relative abundance of key microbes across groups. Statistical analysis: (**D, E**) one-way ANOVA; **(I)** Student's t-test. Significance levels: ^ns^*P* > 0.05; *P < 0.05; **P < 0.01.

Relative abundance analysis showed that Firmicutes and Bacteroidota were the dominant phyla (Figure 6A). At the genus level, the top taxa included *Methanocorpusculum, Rikenellaceae_RC9_gut_group, Treponema, Fibrobacter, Prevotellaceae_UCG-001, NK4A214_group*, Ruminococcus, Methanobrevibacter, Prevotellaceae_UCG-004, and *Lachnospiraceae_UCG-009* (Figure 6B). At the species level, *Fibrobacter_sp_UWH6, Ruminococcus_sp_HUN007, Fibrobacter_sp, Treponema_sp_9AD01, Clostridiales_bacterium_Firm_14, bacterium_P201, Ruminococcus_flavefaciens, Treponema_saccharophilum, bacterium_XPD3003*, and *rumen_bacterium_NK4A65* were most abundant (Figure 6C). Notably, *Rikenellaceae_RC9_gut_group, Treponema*, and *Ruminococcus* were enriched in MG and HG groups at the genus level (Supplementary Table S6), while *Ruminococcus_sp_HUN007, Treponema_sp_9AD01*, and *Fibrobacter_sp* were enriched at the species level (Supplementary Table S7).

Alpha diversity analysis (Shannon and Chao1 indices) revealed significantly higher microbial diversity and richness in HG foals compared with control and LG groups (Figures 6D, E). PCoA based on Bray-Curtis distance showed that PCoA1 and PCoA2 explained 11.75% and 10.50% of total variation, respectively (Figure 6F). Adonis tests indicated significant differences between LG vs. MG (R^2^ = 0.17, *P* = 0.042) and LG vs. HG (R^2^ = 0.17, *P* = 0.038), but not between other groups, confirming distinct microbial communities in MG and HG compared with LG.

LEfSe analysis (LDA score > 3.5) identified six differentially enriched taxa (Figures 6G, H). No significant biomarkers were detected in DG. LG was characterized by f_p-251-o5 and *f_Oscillospiraceae*; MG by *f_Oscillospiraceae*; and HG by *g_Ruminococcus, o_Lachnospirales*, and *f_Lachnospiraceae*. The relative abundances of *g_Ruminococcus, o_Lachnospirales*, and *f_Lachnospiraceae* were significantly higher in HG than MG (Figure 6H), while *f_p-251-o5* was significantly lower in HG than LG (Figure 6I).

### 3.6 Correlation between foal fecal microbiota and plasma amino acid levels after maternal BCAAs supplementation

**Figure 7:**
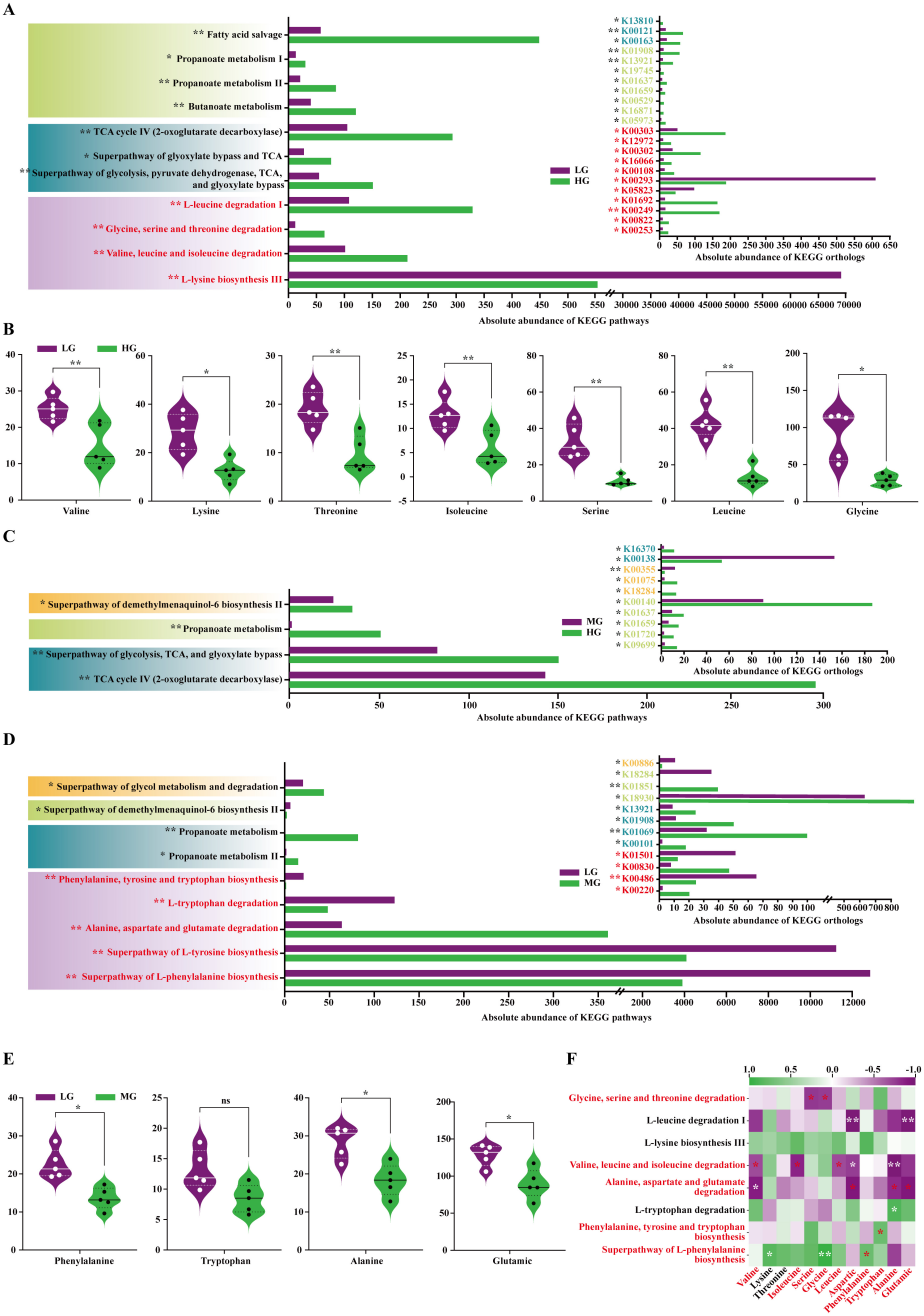
KEGG functional prediction of the foal gut microbiota based on PICRUSt2. **(A)** Differences in KEGG pathway and Ortholog abundance between the HG and LG groups. **(B)** Differences in abundance of relevant amino acids in pathways between the HG and LG groups. **(C)** Differences in KEGG pathway and Ortholog abundance between the HG and MG groups. **(D)** Differences in KEGG pathway and Ortholog abundance between the MG and LG groups. **(E)** Differences in abundance of relevant amino acids in pathways between the MG and LG groups. **(F)** Heatmap of Pearson correlations between significantly different pathways and foal serum amino acid content. All between-group comparisons were performed using Student's t-test. Significance levels: ^ns^*P* > 0.05; *P < 0.05; **P < 0.01.

KEGG functional prediction via PICRUSt2 showed that HG vs. LG differences were mainly enriched in amino acid degradation and synthesis, short-chain fatty acid (SCFA) metabolism, and energy metabolism. Degradation pathways of valine, leucine, isoleucine, and other free amino acids (glycine, serine, threonine) were significantly enriched in HG (*P* < 0.01). SCFA metabolism (*P* < 0.05) and energy metabolism (*P* < 0.05) were also elevated (Figure 7A).

Consistently, plasma levels of valine, leucine, isoleucine, glycine, serine, and threonine were significantly lower in the HG group (*P* < 0.05 or *P* < 0.01; Figure 7B). Analysis of relevant degradative enzymes showed that BCAA degradation enzymes, including K00249 (acyl-CoA dehydrogenase, ACADM), K00253 (isovaleryl-CoA dehydrogenase, IVD), K05605 (3-hydroxyisobutyryl-CoA hydrolase, HIBCH) and other amino acid degradation enzymes, such as K00529 (3-phenylpropionate dioxygenase reductase, hcaD), K01692 (enoyl-CoA hydratase, paaF), K00822 (beta-alanine-pyruvate transaminase), K01028 (3-oxoacid CoA-transferase subunit A, scoA), K12972 (glyoxylate/hydroxypyruvate reductase, ghrA), K16066 (3-hydroxy acid dehydrogenase, ydfG) were significantly upregulated in HG (*P* < 0.05). In contrast, the lysine synthesis pathway was less abundant in HG (*P* < 0.01), and lysine synthesis enzymes K00293 (saccharopine dehydrogenase, LYS9) and K05823 (N-acetyldiaminopimelate deacetylase, dapL) were downregulated (*P* < 0.05; Figure 7A).

Differences between HG and MG were mainly related to SCFA and energy metabolism, with pathway abundances significantly higher in HG (*P* < 0.01; Figure 7C). Between MG and LG, microbial functional differences involved non-BCAA degradation and synthesis, and SCFA metabolism. Tryptophan, alanine, aspartate, and glutamate degradation pathways were significantly more abundant in MG (*P* < 0.01; Figure 7D). Plasma levels of these amino acids (except tryptophan, *P* > 0.05) were lower in MG (*P* < 0.05; Figure 7E). The degradation enzymes K00830 (alanine-glyoxylate transaminase/serine-glyoxylate transaminase/serine-pyruvate transaminase, AGXT) and K00220 (cyclohexadienyl/prephenate dehydrogenase, tyrC) were upregulated in the MG group (*P* < 0.05). Synthesis pathways for phenylalanine, tyrosine, and tryptophan were less abundant in MG (*P* < 0.01), and synthesis enzymes K00486 (kynurenine 3-monooxygenase, KMO) and K01501 (nitrilase) were downregulated (*P* < 0.05). Propionate metabolism and enzyme activity were higher in MG (*P* < 0.05; Figure 7D). Microbial amino acid degradation and synthesis pathways were significantly correlated with plasma amino acids (|*r*| > 0.70, *P* < 0.05): degradation pathways were negatively correlated, and synthesis pathways were positively correlated (Figure 7F).

## 4 Discussion

Amino acids serve as the fundamental building blocks of proteins and participate extensively in diverse metabolic pathways essential for maintaining physiological homeostasis (Akers, 2016). In mammals, the demand for amino acids increases substantially during lactation to support milk synthesis in the mammary gland (Blackburn et al., 1989). Among the essential amino acids, branched-chain amino acids (BCAAs) have attracted significant research interest in recent years. Studies indicate that BCAAs undergo extensive catabolism in mammary tissue, providing amino groups for the synthesis of other amino acids such as glutamate and glutamine (Wu et al., 2006; Lei et al., 2012), which are crucial for neonatal growth and development of the gastrointestinal tract (Rezaei et al., 2016). Growing evidence suggests that BCAAs play regulatory roles in mammary metabolism. For instance, leucine activates the mTOR signaling pathway, thereby promoting milk fat and protein synthesis in bovine and porcine mammary epithelial cells (Bo and Fujii, 2024; Green et al., 2024). Our findings further demonstrate that supplementing lactating mares with BCAAs enhances milk fat yield in a dose-dependent manner, with an effective threshold dose of 76 g/d. Notably, changes in growth hormone (GH) and progesterone levels exhibited temporal and dose-response patterns closely paralleling milk fat synthesis.

GH is a key regulator of mammary development in periparturient dairy cows. Exogenous GH administration significantly increases mammary parenchymal tissue and improves lactational performance (Jiang et al., 2025). Previous studies have shown that elevated GH, in coordination with IGF-1, promotes milk fat synthesis by phosphorylating and increasing the abundance and activity of acetyl-CoA carboxylase (ACC), thereby activating the fatty acid synthesis pathway in the mammary gland and enhancing milk fat secretion efficiency (Kim et al., 1997). Progesterone, an ovarian steroid hormone, plays an essential role in alveolar morphogenesis. Although elevated progesterone levels inhibit lactogenesis (Jiang et al., 2025; Basu et al., 2025), physiological concentrations support the formation and maintenance of lobuloalveolar structures and delay ductal involution during lactation (Blackburn et al., 1989).

The nutritional and metabolic regulatory effects of BCAAs are further reflected in the altered metabolic profiles (Bo and Fujii, 2024; Ying et al., 2025). Untargeted metabolomics analysis in this study revealed clear separation of treatment groups through multivariate statistical methods (PCA, OPLS-DA), with organic acids and derivatives representing the most significantly affected class of differentially expressed metabolites (DEMs). Pathway enrichment analysis indicated that BCAA intervention significantly influenced branched-chain amino acid metabolism, lipid metabolism, energy metabolism, and carbohydrate metabolism. Key pathways involved included alanine, aspartate and glutamate metabolism; glycine, serine and threonine metabolism; and valine, leucine and isoleucine biosynthesis. These results suggest that BCAAs may contribute to metabolic regulation by providing exogenous EAAs to hepatic and mammary tissues (Zhao et al., 2024). Furthermore, milk from the high-dose BCAA group showed significantly elevated levels of leucine and valine compared to controls. As a potent activator of the mTORC1 pathway, leucine directly stimulates mammary lipid synthesis (Zhao et al., 2024). The steroid hormone biosynthesis pathway was also identified as significantly affected, with progesterone content substantially increased in the high-dose group while estrogen levels remained unchanged. Correlation analysis revealed significant associations between valine, leucine, and progesterone levels with key metabolites in these pathways, suggesting that high-dose BCAAs supplementation promotes milk fat yield through coordinated regulation of BCAA metabolism and progesterone synthesis. Moreover, leucine and valine were significantly positively correlated with tricarboxylic acid (TCA) cycle intermediates, such as alpha-ketoglutarate and succinate, as well as glutamine-related metabolites, including D-glutamine and N-alpha-acetylornithine. These correlations suggest that high-dose BCAA supplementation may enhance mammary cellular energy metabolism and nitrogen flux. Specifically, BCAAs can be transaminated and converted into TCA cycle intermediates, providing additional carbon skeletons that feed into the cycle, thereby increasing the production of ATP and reducing equivalents (NADPH). The elevated NADPH and acetyl-CoA generated from this metabolic flux serve as essential substrates for de novo fatty acid synthesis in mammary epithelial cells. Concurrently, glutamine-related metabolites contribute to nitrogen homeostasis and can be converted to alpha-ketoglutarate, further supporting TCA cycle activity and maintaining redox balance (Zhao et al., 2024). Together, these metabolic adjustments provide both the energy and the building blocks required for enhanced milk fat synthesis, offering a mechanistic explanation for the observed increases in milk fat yield.

Accumulating evidence indicates that BCAAs function not only as protein synthesis substrates but also as signaling molecules and hormone secretion regulators, playing key roles in stimulating insulin (INS), growth hormone (GH), and insulin-like growth factor-1 (IGF-1) release (Yan et al., 2024). Our study further demonstrates that foals consuming milk from mares receiving medium and high BCAA doses exhibited significant physiological alterations. The most pronounced response was the activation of GH, IGF-1, and INS: by day 60, serum levels of these hormones were significantly elevated in these foals, showing a clear dose-dependence. This provides direct evidence that maternal nutrition influences offspring development through milk composition. Notably, our previous research established that medium-dose BCAA supplementation significantly promotes body height and length gains in foals (Ren et al., 2025).

Blood urea nitrogen (BUN) levels displayed a nonlinear response, being lowest in the medium-dose group and highest in the high-dose group. Interestingly, despite higher BCAA intake through milk, serum concentrations of numerous amino acids, including BCAAs (Val, Leu, Ile) and multiple non-BCAAs (e.g., Arg, Lys, Pro, Asp, Glu, Thr, Ala, Phe, Trp, Tyr, Ser, Gly, Met, His, Orn) were significantly reduced in the HG group. The MG group also showed decreased levels of BCAAs and several non-BCAAs (Met, Gly, Ser, Phe, Lys, Trp, Ala, Glu, Pro, Thr). This phenomenon likely reflects metabolic reprogramming under hormonal regulation. Elevated IGF-1 (Yan et al., 2024; Maimaituxun et al., 2024), GH (Maimaituxun et al., 2024; Baron et al., 1993), and INS (Baron et al., 1993; Vary and Lynch, 2007) levels in rapidly growing foals collectively enhance tissue perfusion and accelerate amino acid transport into skeletal muscle. Simultaneously, leucine activates the mTOR signaling pathway to promote eIF4E-eIF4G complex formation and protein synthesis (Appuhamy et al., 2012), while also inhibiting BCKDH kinase and activating the branched-chain α-keto acid dehydrogenase complex to enhance BCAA catabolism (Li et al., 2009; Lynch and Adams, 2014; Xu et al., 2022). These adaptations collectively support intensive growth and protein deposition in foals, leading to reduced serum amino acid concentrations and increased urea nitrogen production (Xu et al., 2022). The elevated BUN in the HG group may indicate reduced nitrogen utilization efficiency and increased excretory burden, whereas the lowest BUN in the MG group suggests optimal nitrogen retention and utilization. However, the broad reduction in amino acids, particularly non-BCAAs, cannot be fully explained by enhanced tissue uptake alone. Residual amino acids that are not immediately utilized by the host can be metabolized by the intestinal microbiota, generating short-chain fatty acids and other metabolites that, in turn, modulate host nutrient absorption, energy metabolism, and anabolic signaling. Recent studies highlight the crucial role of the gut microbiota in maintaining host amino acid homeostasis (Heianza et al., 2019). In this study, maternal BCAA supplementation significantly altered foal gut microbiota structure and diversity, strongly implying that functional changes in microbial metabolism contributed to the observed decline in serum amino acids. The microbiota may enhance amino acid utilization and conversion, thereby modulating the host's circulating amino acid pool.

PICRUSt2 functional prediction analysis of foal fecal microbiota revealed that maternal BCAA supplementation significantly remodeled microbial metabolic function, particularly enhancing the degradation capacity for branched-chain and other amino acids. Gut microbiota exhibit high amino acid degradation rates, preferentially utilizing substrates such as lysine, arginine, glycine, and BCAAs (leucine, valine, isoleucine) (Barker, 1981; Li et al., 2024; Sinha et al., 2025; Tobón-Cornejo et al., 2025). This degradation generates various metabolites including ammonia (Wu et al., 2021), short-chain fatty acids (SCFAs e.g., acetate, propionate, butyrate) (Li et al., 2024; Sinha et al., 2025; Qiao et al., 2022), and branched-chain fatty acids (e.g., isobutyrate, isovalerate, valerate) (Dai et al., 2021). These metabolites serve not only as energy sources but also modulate host intestinal epithelial signaling and regulate bacterial gene expression (Hyland et al., 2022; Liu et al., 2022), ultimately influencing the production of enzymes involved in amino acid metabolism (Nemet et al., 2023; Hsu et al., 2024). In the present study, HG foals showed significantly upregulated abundances of valine, leucine, and isoleucine degradation pathways and associated enzyme genes (e.g., ACADM, IVD, HIBCH). Simultaneously, degradation pathways for glycine, serine, and threonine in the HG group, and for tryptophan, alanine, aspartate, and glutamate in the MG group, were significantly enhanced. Conversely, the lysine synthesis pathway in the HG group and phenylalanine, tyrosine, and tryptophan synthesis pathways in the MG group were significantly reduced, indicating a functional shift toward microbial amino acid degradation. Importantly, these microbial functional changes strongly correlated with host serum amino acid levels: microbial degradation pathway abundances negatively correlated with corresponding serum amino acids, while synthesis pathway abundances showed positive correlations. These results establish that gut microbial amino acid metabolism constitutes a central mechanism regulating systemic amino acid homeostasis in the host. Additionally, BCAAs supplementation increased the abundance of microbial pathways for SCFA synthesis (including acetate, propionate, and butyrate). SCFAs play vital roles in maintaining intestinal epithelial health (Liu et al., 2024) and regulating immune and inflammatory responses (Mann et al., 2024; Wang Q. et al., 2025), potentially providing additional metabolic benefits for growing foals.

This study provides an integrated analysis of the complete mechanistic pathway through which maternal BCAAs supplementation influences lactational performance and offspring growth. The findings advance our theoretical understanding of equine lactation physiology and host-microbiota interactions, while also offering a scientific basis for optimizing mare nutritional strategies to enhance foal growth performance. However, certain limitations should be acknowledged: the sample size may limit statistical power for low-abundance measures, and functional inference based on 16S rRNA sequencing and PICRUSt2 has inherent constraints. Future studies should incorporate finer dose gradients to more precisely determine optimal BCAAs supplementation levels. Furthermore, approaches such as fecal microbiota transplantation (FMT), combined with targeted quantification of key metabolites (e.g., SCFAs, TCA cycle intermediates, hormones) and direct assays of relevant metabolic enzyme activities, will be valuable to validate causal relationships of microbial functions and further elucidate the molecular mechanisms and practical applications of maternal BCAAs supplementation.

## 5 Conclusion

This study employed an integrated multi-omics approach to systematically elucidate the pathway through which BCAAs regulate milk fat synthesis in lactating mares and offspring growth via the “milk compounds-fecal microbiota-host metabolism” axis. We not only identified the optimal BCAAs supplementation dosage in the mare-foal model, but also revealed that remodeling of gut microbial amino acid metabolism in foals is a central mechanism mediating the intergenerational effects under medium- and high-dose supplementation regimens. These findings provide a important theoretical foundation and practical strategies for improving milk quality and promoting healthy growth of suckling foals through maternal nutritional intervention.

## Data Availability

The original contributions presented in the study are included in the article/Supplementary material, further inquiries can be directed to the corresponding author.
