# Supplementary material for: BCAA supplementation enhances milk fat synthesis in Yili mares and promotes foal growth through remodeling of intestinal amino acid metabolism
Source: Front Microbiol. 2025 Nov 6;16:1699614. doi: 10.3389/fmicb.2025.1699614 (PMC12633753; doi:10.3389/fmicb.2025.1699614)
Supplement: Supplementary file 1 [file Table_1.docx]

Supplementary Material

# Supplementary Tables

# Supplementary Table 1. Results of the mixed linear model for mare indicators under different BCAA dosage treatments.

| **Main Effect** | **Sum Sq** | **Mean Sq** | **NumDF** | **DenDF** | **F value** | **Pr(>F)** |
| --- | --- | --- | --- | --- | --- | --- |
| GH: Treatment | 236.57 | 78.86 | 3.00 | 32.00 | 14.57 | 0.00 |
| GH: Time | 58.11 | 58.11 | 1.00 | 32.00 | 10.74 | 0.00 |
| GH: Treatment × Time | 131.52 | 43.84 | 3.00 | 32.00 | 8.10 | 0.00 |
| MFP: Treatment | 220470.71 | 73490.24 | 3.00 | 16.00 | 23.46 | 0.00 |
| MFP: Time | 1005217.95 | 251304.49 | 4.00 | 64.00 | 80.21 | 0.00 |
| MFP: Treatment × Time | 288368.97 | 24030.75 | 12.00 | 64.00 | 7.67 | 0.00 |
| PROG: Treatment | 616589.16 | 205529.72 | 3.00 | 32.00 | 7.41 | 0.00 |
| PROG: Time | 6067397.34 | 6067397.34 | 1.00 | 32.00 | 218.84 | 0.00 |
| PROG: Treatment × Time | 346571.54 | 115523.85 | 3.00 | 32.00 | 4.17 | 0.01 |

# Supplementary Table 2. Results of post-hoc Tukey’s HSD tests for various parameters in mares treated with different dosages of BCAAs.

| **Milk fat production contrasts** | **Time** | **Estimate** | **SE** | **DF** | **Tratio** | **Pvalue** |
| --- | --- | --- | --- | --- | --- | --- |
| Treatment0 - Treatment38 | 0 | -14.07 | 37.57 | 76.16 | -0.37 | 0.98 |
| Treatment0 - Treatment76 | 0 | -20.47 | 37.57 | 76.16 | -0.54 | 0.95 |
| Treatment0 - Treatment114 | 0 | -40.56 | 37.57 | 76.16 | -1.08 | 0.7 |
| Treatment38 - Treatment76 | 0 | -6.39 | 37.57 | 76.16 | -0.17 | 1.00 |
| Treatment38 - Treatment114 | 0 | -26.49 | 37.57 | 76.16 | -0.71 | 0.89 |
| Treatment76 - Treatment114 | 0 | -20.09 | 37.57 | 76.16 | -0.53 | 0.95 |
| Treatment0 - Treatment38 | 15 | -42.97 | 37.57 | 76.16 | -1.14 | 0.66 |
| Treatment0 - Treatment76 | 15 | -37.44 | 37.57 | 76.16 | -1 | 0.75 |
| Treatment0 - Treatment114 | 15 | -61.08 | 37.57 | 76.16 | -1.63 | 0.37 |
| Treatment38 - Treatment76 | 15 | 5.52 | 37.57 | 76.16 | 0.15 | 1.00 |
| Treatment38 - Treatment114 | 15 | -18.11 | 37.57 | 76.16 | -0.48 | 0.96 |
| Treatment76 - Treatment114 | 15 | -23.64 | 37.57 | 76.16 | -0.63 | 0.92 |
| Treatment0 - Treatment38 | 30 | -23.23 | 37.57 | 76.16 | -0.62 | 0.93 |
| Treatment0 - Treatment76 | 30 | 2.22 | 37.57 | 76.16 | 0.06 | 1.00 |
| Treatment0 - Treatment114 | 30 | -48.68 | 37.57 | 76.16 | -1.30 | 0.57 |
| Treatment38 - Treatment76 | 30 | 25.46 | 37.57 | 76.16 | 0.68 | 0.91 |
| Treatment38 - Treatment114 | 30 | -25.45 | 37.57 | 76.16 | -0.68 | 0.91 |
| Treatment76 - Treatment114 | 30 | -50.91 | 37.57 | 76.16 | -1.35 | 0.53 |
| Treatment0 - Treatment38 | 45 | -301.38 | 37.57 | 76.16 | -8.02 | 0.00 |
| Treatment0 - Treatment76 | 45 | -200.73 | 37.57 | 76.16 | -5.34 | 0.00 |
| Treatment0 - Treatment114 | 45 | -372.11 | 37.57 | 76.16 | -9.9 | 0.00 |
| Treatment38 - Treatment76 | 45 | 100.66 | 37.57 | 76.16 | 2.68 | 0.04 |
| Treatment38 - Treatment114 | 45 | -70.73 | 37.57 | 76.16 | -1.88 | 0.24 |
| Treatment76 - Treatment114 | 45 | -171.38 | 37.57 | 76.16 | -4.56 | 0.00 |
| Treatment0 - Treatment38 | 60 | -209.06 | 37.57 | 76.16 | -5.56 | 0.00 |
| Treatment0 - Treatment76 | 60 | -127.66 | 37.57 | 76.16 | -3.4 | 0.01 |
| Treatment0 - Treatment114 | 60 | -292.52 | 37.57 | 76.16 | -7.79 | 0.00 |
| Treatment38 - Treatment76 | 60 | 81.41 | 37.57 | 76.16 | 2.17 | 0.14 |
| Treatment38 - Treatment114 | 60 | -83.46 | 37.57 | 76.16 | -2.22 | 0.13 |
| Treatment76 - Treatment114 | 60 | -164.86 | 37.57 | 76.16 | -4.39 | 0.00 |
| **GH contrasts** | **Time** | **Estimate** | **SE** | **DF** | **Tratio** | **Pvalue** |
| Treatment0 - Treatment38 | 0 | 0.84 | 1.47 | 32 | 0.57 | 0.94 |
| Treatment0 - Treatment76 | 0 | -1.82 | 1.47 | 32 | -1.23 | 0.61 |
| Treatment0 - Treatment114 | 0 | -1.42 | 1.47 | 32 | -0.96 | 0.77 |
| Treatment38 - Treatment76 | 0 | -2.65 | 1.47 | 32 | -1.8 | 0.29 |
| Treatment38 - Treatment114 | 0 | -2.25 | 1.47 | 32 | -1.53 | 0.43 |
| Treatment76 - Treatment114 | 0 | 0.4 | 1.47 | 32 | 0.27 | 0.99 |
| Treatment0 - Treatment38 | 60 | -3.63 | 1.47 | 32 | -2.46 | 0.09 |
| Treatment0 - Treatment76 | 60 | -5.19 | 1.47 | 32 | -3.53 | 0.01 |
| Treatment0 - Treatment114 | 60 | -11.49 | 1.47 | 32 | -7.81 | 0.00 |
| Treatment38 - Treatment76 | 60 | -1.56 | 1.47 | 32 | -1.06 | 0.71 |
| Treatment38 - Treatment114 | 60 | -7.87 | 1.47 | 32 | -5.35 | 0.00 |
| Treatment76 - Treatment114 | 60 | -6.3 | 1.47 | 32 | -4.28 | 0.00 |
| **PROG contrasts** | **Time** | **Estimate** | **SE** | **DF** | **Tratio** | **Pvalue** |
| Treatment0 - Treatment38 | 0 | -59.57 | 105.31 | 32 | -0.57 | 0.94 |
| Treatment0 - Treatment76 | 0 | -57.01 | 105.31 | 32 | -0.54 | 0.95 |
| Treatment0 - Treatment114 | 0 | -116.11 | 105.31 | 32 | -1.1 | 0.69 |
| Treatment38 - Treatment76 | 0 | 2.55 | 105.31 | 32 | 0.02 | 1.00 |
| Treatment38 - Treatment114 | 0 | -56.54 | 105.31 | 32 | -0.54 | 0.95 |
| Treatment76 - Treatment114 | 0 | -59.09 | 105.31 | 32 | -0.56 | 0.94 |
| Treatment0 - Treatment38 | 60 | -517.24 | 105.31 | 32 | -4.91 | 0.00 |
| Treatment0 - Treatment76 | 60 | -138.18 | 105.31 | 32 | -1.31 | 0.56 |
| Treatment0 - Treatment114 | 60 | -456.37 | 105.31 | 32 | -4.33 | 0.00 |
| Treatment38 - Treatment76 | 60 | 379.07 | 105.31 | 32 | 3.6 | 0.01 |
| Treatment38 - Treatment114 | 60 | 60.87 | 105.31 | 32 | 0.58 | 0.94 |
| Treatment76 - Treatment114 | 60 | -318.19 | 105.31 | 32 | -3.02 | 0.02 |

# Supplementary Table 3. Classification of key metabolites in mare milk.

| **Index** | **Super class** | **Class** | **Sub class** | **ID** |
| --- | --- | --- | --- | --- |
| Alpha-ketoglutarate | Organic acids and derivatives | Keto acids and derivatives | Gamma-keto acids and derivatives | C00026 |
| Succinate | Organic acids and derivatives | Carboxylic acids and derivatives | Dicarboxylic acids and derivatives | C00042 |
| L-Histidine | Organic acids and derivatives | Carboxylic acids and derivatives | Amino acids, peptides, and analogues | C00135 |
| DL-threonine | / | / | / | C00188 |
| Histamine | Organic nitrogen compounds | Organonitrogen compounds | Amines | C00388 |
| N-alpha-acetyl-l-ornithine | / | / | / | C00437 |
| Pyruvaldehyde | Organic oxygen compounds | Organooxygen compounds | Carbonyl compounds | C00546 |
| Betaine | Organic acids and derivatives | Carboxylic acids and derivatives | Amino acids, peptides, and analogues | C00719 |
| D-glutamine | Organic acids and derivatives | Carboxylic acids and derivatives | Amino acids, peptides, and analogues | C00819 |
| 3-Methylhistidine | Organic acids and derivatives | Carboxylic acids and derivatives | Amino acids, peptides, and analogues | C01152 |
| Anserine | Organic acids and derivatives | Peptidomimetics | Hybrid peptides | C01262 |
| 4-androsten-11.beta.-ol-3,17-dione | Organoheterocyclic compounds | Oxazinanes | Morpholines | C05284 |
| Phenaceturic acid | Organic acids and derivatives | Carboxylic acids and derivatives | Amino acids, peptides, and analogues | C05598 |
| 1,5-diphenylcarbohydrazide | / | / | / | C06560 |
| Cholesteryl sulfate | Lipids and lipid-like molecules | Steroids and steroid derivatives | Cholestane steroids | C18043 |

**Supplementary Table 4.** Results of the mixed linear model for foal parameters in response to different BCAA dosage treatments.

| **Main Effect** | **Sum Sq** | **Mean Sq** | **NumDF** | **DenDF** | **F value** | **Pr(>F)** |
| --- | --- | --- | --- | --- | --- | --- |
| GH: Treatment | 3.85 | 1.28 | 3.00 | 16.00 | 3.20 | 0.04 |
| GH: Time | 7.79 | 7.79 | 1.00 | 16.00 | 19.40 | 0.00 |
| GH: Treatment × Time | 5.49 | 1.83 | 3.00 | 16.00 | 4.56 | 0.02 |
| IGF-1: Treatment | 2480.78 | 826.93 | 3.00 | 32.00 | 5.39 | 0.00 |
| IGF-1: Time | 1740.68 | 1740.68 | 1.00 | 32.00 | 11.35 | 0.00 |
| IGF-1: Treatment × Time | 1055.96 | 351.99 | 3.00 | 32.00 | 2.30 | 0.05 |
| BUN: Treatment | 8.39 | 2.80 | 3.00 | 16.00 | 6.81 | 0.00 |
| BUN: Time | 12.63 | 12.63 | 1.00 | 16.00 | 30.77 | 0.00 |
| BUN: Treatment × Time | 3.21 | 1.07 | 3.00 | 16.00 | 2.61 | 0.04 |
| INS: Treatment | 70.74 | 23.58 | 3.00 | 32.00 | 22.64 | 0.00 |
| INS: Time | 4.03 | 4.03 | 1.00 | 32.00 | 3.87 | 0.04 |
| INS: Treatment × Time | 46.25 | 15.42 | 3.00 | 32.00 | 14.80 | 0.00 |

**Supplementary Table 5.** Results of the mixed linear model for foal parameters in response to different BCAA dosage treatments.

| **GH contrast** | **Time** | **Estimate** | **SE** | **DF** | **Tratio** | **Pvalue** |
| --- | --- | --- | --- | --- | --- | --- |
| Treatment0 - Treatment38 | 0.00 | 0.58 | 0.46 | 30.39 | 1.28 | 0.58 |
| Treatment0 - Treatment76 | 0.00 | 0.91 | 0.46 | 30.39 | 1.99 | 0.21 |
| Treatment0 - Treatment114 | 0.00 | 0.30 | 0.46 | 30.39 | 0.66 | 0.91 |
| Treatment38 - Treatment76 | 0.00 | 0.32 | 0.46 | 30.39 | 0.71 | 0.89 |
| Treatment38 - Treatment114 | 0.00 | -0.28 | 0.46 | 30.39 | -0.62 | 0.93 |
| Treatment76 - Treatment114 | 0.00 | -0.61 | 0.46 | 30.39 | -1.33 | 0.55 |
| Treatment0 - Treatment38 | 60.00 | 0.34 | 0.46 | 30.39 | 0.74 | 0.88 |
| Treatment0 - Treatment76 | 60.00 | -0.43 | 0.46 | 30.39 | -0.94 | 0.79 |
| Treatment0 - Treatment114 | 60.00 | -1.48 | 0.46 | 30.39 | -3.25 | 0.01 |
| Treatment38 - Treatment76 | 60.00 | -0.76 | 0.46 | 30.39 | -1.67 | 0.36 |
| Treatment38 - Treatment114 | 60.00 | -1.82 | 0.46 | 30.39 | -3.98 | 0.00 |
| Treatment76 - Treatment114 | 60.00 | -1.06 | 0.46 | 30.39 | -2.31 | 0.12 |
| **IGF-1 contrast** | **Time** | **Estimate** | **SE** | **DF** | **Tratio** | **Pvalue** |
| Treatment0 - Treatment38 | 0.00 | 3.33 | 7.83 | 32.00 | 0.42 | 0.97 |
| Treatment0 - Treatment76 | 0.00 | -1.79 | 7.83 | 32.00 | -0.23 | 1.00 |
| Treatment0 - Treatment114 | 0.00 | -5.19 | 7.83 | 32.00 | -0.66 | 0.91 |
| Treatment38 - Treatment76 | 0.00 | -5.12 | 7.83 | 32.00 | -0.65 | 0.91 |
| Treatment38 - Treatment114 | 0.00 | -8.52 | 7.83 | 32.00 | -1.09 | 0.70 |
| Treatment76 - Treatment114 | 0.00 | -3.40 | 7.83 | 32.00 | -0.43 | 0.97 |
| Treatment0 - Treatment38 | 60.00 | -2.35 | 7.83 | 32.00 | -0.30 | 0.99 |
| Treatment0 - Treatment76 | 60.00 | -14.58 | 7.83 | 32.00 | -1.86 | 0.26 |
| Treatment0 - Treatment114 | 60.00 | -32.65 | 7.83 | 32.00 | -4.17 | 0.00 |
| Treatment38 - Treatment76 | 60.00 | -12.23 | 7.83 | 32.00 | -1.56 | 0.41 |
| Treatment38 - Treatment114 | 60.00 | -30.30 | 7.83 | 32.00 | -3.87 | 0.00 |
| Treatment76 - Treatment114 | 60.00 | -18.06 | 7.83 | 32.00 | -2.31 | 0.12 |
| **BUN contrast** | **Time** | **Estimate** | **SE** | **DF** | **Tratio** | **Pvalue** |
| Treatment0 - Treatment38 | 0.00 | 0.42 | 0.48 | 29.60 | 0.87 | 0.82 |
| Treatment0 - Treatment76 | 0.00 | 0.87 | 0.48 | 29.60 | 1.82 | 0.28 |
| Treatment0 - Treatment114 | 0.00 | 0.85 | 0.48 | 29.60 | 1.77 | 0.31 |
| Treatment38 - Treatment76 | 0.00 | 0.45 | 0.48 | 29.60 | 0.95 | 0.78 |
| Treatment38 - Treatment114 | 0.00 | 0.43 | 0.48 | 29.60 | 0.90 | 0.81 |
| Treatment76 - Treatment114 | 0.00 | -0.02 | 0.48 | 29.60 | -0.05 | 1.00 |
| Treatment0 - Treatment38 | 60.00 | 0.20 | 0.48 | 29.60 | 0.41 | 0.98 |
| Treatment0 - Treatment76 | 60.00 | 1.75 | 0.48 | 29.60 | 3.66 | 0.01 |
| Treatment0 - Treatment114 | 60.00 | 1.96 | 0.48 | 29.60 | 4.10 | 0.00 |
| Treatment38 - Treatment76 | 60.00 | 1.56 | 0.48 | 29.60 | 3.25 | 0.01 |
| Treatment38 - Treatment114 | 60.00 | 1.77 | 0.48 | 29.60 | 3.69 | 0.00 |
| Treatment76 - Treatment114 | 60.00 | 0.21 | 0.48 | 29.60 | 0.44 | 0.97 |
| **INS contrast** | **Time** | **Estimate** | **SE** | **DF** | **Tratio** | **Pvalue** |
| Treatment0 - Treatment38 | 0.00 | 0.01 | 0.65 | 32.00 | 0.02 | 1.00 |
| Treatment0 - Treatment76 | 0.00 | 0.41 | 0.65 | 32.00 | 0.63 | 0.92 |
| Treatment0 - Treatment114 | 0.00 | -1.18 | 0.65 | 32.00 | -1.83 | 0.28 |
| Treatment38 - Treatment76 | 0.00 | 0.40 | 0.65 | 32.00 | 0.61 | 0.93 |
| Treatment38 - Treatment114 | 0.00 | -1.20 | 0.65 | 32.00 | -1.85 | 0.27 |
| Treatment76 - Treatment114 | 0.00 | -1.59 | 0.65 | 32.00 | -2.47 | 0.09 |
| Treatment0 - Treatment38 | 60.00 | 0.02 | 0.65 | 32.00 | 0.04 | 1.00 |
| Treatment0 - Treatment76 | 60.00 | -4.26 | 0.65 | 32.00 | -6.60 | 0.00 |
| Treatment0 - Treatment114 | 60.00 | -5.03 | 0.65 | 32.00 | -7.79 | 0.00 |
| Treatment38 - Treatment76 | 60.00 | -4.28 | 0.65 | 32.00 | -6.63 | 0.00 |
| Treatment38 - Treatment114 | 60.00 | -5.05 | 0.65 | 32.00 | -7.83 | 0.00 |
| Treatment76 - Treatment114 | 60.00 | -0.77 | 0.65 | 32.00 | -1.20 | 0.63 |

**Supplementary Table 6.** The relative abundance of the Top 10 bacterial genera in the fecal microbiota of foals.

| **Group** | **Methanocorpusculum** | **Rikenellaceae_RC9_gut_group** | **Treponema** | **Fibrobacter** | **Prevotellaceae_UCG-001** | **NK4A214_group** | **Ruminococcus** | **Methanobrevibacter** | **Prevotellaceae_UCG-004** | **Lachnospiraceae_UCG-009** | **Others** |
| --- | --- | --- | --- | --- | --- | --- | --- | --- | --- | --- | --- |
| DG | 0.01 | 0.11 | 0.05 | 0.03 | 0.03 | 0.03 | 0.03 | 0.00 | 0.02 | 0.02 | 0.66 |
| LG | 0.06 | 0.09 | 0.06 | 0.05 | 0.04 | 0.03 | 0.02 | 0.01 | 0.01 | 0.02 | 0.60 |
| HG | 0.01 | 0.14 | 0.06 | 0.03 | 0.03 | 0.03 | 0.04 | 0.01 | 0.03 | 0.02 | 0.61 |
| MG | 0.01 | 0.13 | 0.09 | 0.04 | 0.04 | 0.02 | 0.04 | 0.00 | 0.02 | 0.03 | 0.60 |

**Supplementary Table 7.** The relative abundance of the Top 10 bacterial species in the fecal microbiota of foals.

| **Group** | **Fibrobacter_sp_UWH6** | **Ruminococcus_sp_HUN007** | **Fibrobacter_sp** | **Treponema_sp_9AD01** | **Clostridiales_bacterium_Firm_14** | **bacterium_P201** | **Ruminococcus_flavefaciens** | **Treponema_saccharophilum** | **bacterium_XPD3003** | **rumen_bacterium_NK4A65** | **Others** |
| --- | --- | --- | --- | --- | --- | --- | --- | --- | --- | --- | --- |
| DG | 0.003 | 0.009 | 0.003 | 0.004 | 0.001 | 0.000 | 0.001 | 0.000 | 0.001 | 0.001 | 0.976 |
| LG | 0.020 | 0.011 | 0.006 | 0.003 | 0.000 | 0.000 | 0.002 | 0.001 | 0.000 | 0.001 | 0.957 |
| HG | 0.005 | 0.015 | 0.005 | 0.009 | 0.001 | 0.001 | 0.001 | 0.000 | 0.001 | 0.001 | 0.963 |
| MG | 0.002 | 0.019 | 0.008 | 0.006 | 0.002 | 0.000 | 0.003 | 0.001 | 0.002 | 0.001 | 0.956 |
